# A case-report on diverticulitis misdiagnosed as tubo-ovarian abscess

**DOI:** 10.1016/j.amsu.2021.103049

**Published:** 2021-11-10

**Authors:** Seyyedeh Neda Kazemi, Masoomeh Raoufi, Majid Samsami, Hamidreza Didar, Haniye Najafiarab

**Affiliations:** a Preventative Gynecology Research Center, Shahid Beheshti University of Medical Sciences, Tehran, Iran; b Department of Obstetrics and Gynecology, Endocrinology and Female Infertility Unit, Tehran University of Medical Sciences, Tehran, Iran; c Department of Radiology, School of Medicine, Shahid Beheshti University of Medical Sciences, Tehran, Iran; d Department of General Surgery, School of Medicine, Imam Hossein Hospital, Shahid Beheshti University of Medical Sciences, Tehran, Iran; e Student of Research Committee, Shahid Beheshti University of Medical Sciences, Tehran, Iran

**Keywords:** TOA, Gastrointestinal, Diverticulitis, Antibiotic, Genital, TOA, Tubo-ovarian abscesses, PID, Pelvic inflammatory disease, CT, computed tomography

## Abstract

**Introduction and importance:**

Tubo-ovarian abscesses (TOA) is presented with multiple clinical manifestations including gastrointestinal findings.

**Case presentation:**

Herein, we present a case of complicated diverticulitis that was misdiagnosed as TOA, owing to overlapping genital involvement.

**Clinical discussion:**

Imaging could be misleading in these patients as a result of severe inflammation of intestines and surrounding organs.

**Conclusion:**

Patients who do not respond to antibiotic treatment should be suspected of other gastrointestinal pathologies such as diverticulitis and should be evaluated accordingly.

## Highlights

• Concomitant presentation of diverticulitis and Tubo-ovarian abscesses (TOA) has been rarely reported.
• Diverticulitis can also be complicated, indicated by the involvement of other organs such as ovaries and fallopian tube.
• In such cases, pathological evaluation, following the surgical intervention can indicate the exact disease.

## 1 Introduction

**Fig. 1:**
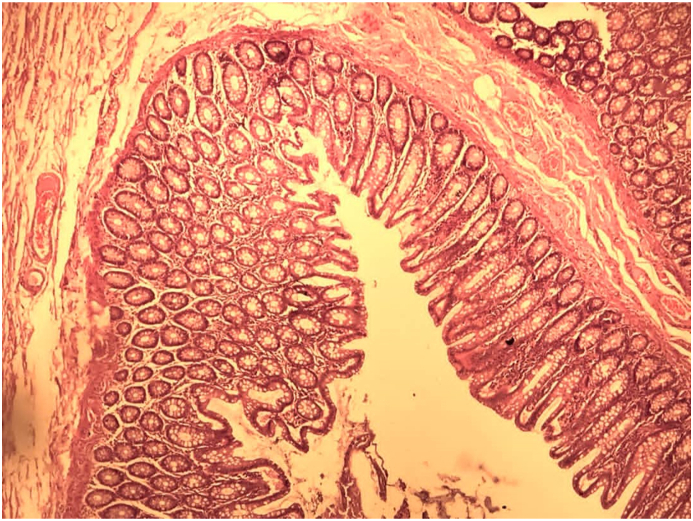
Photomicrograph of colon diverticula showing central lumen with surrounding mucosa (H&E straining, X100).

**Fig. 2:**
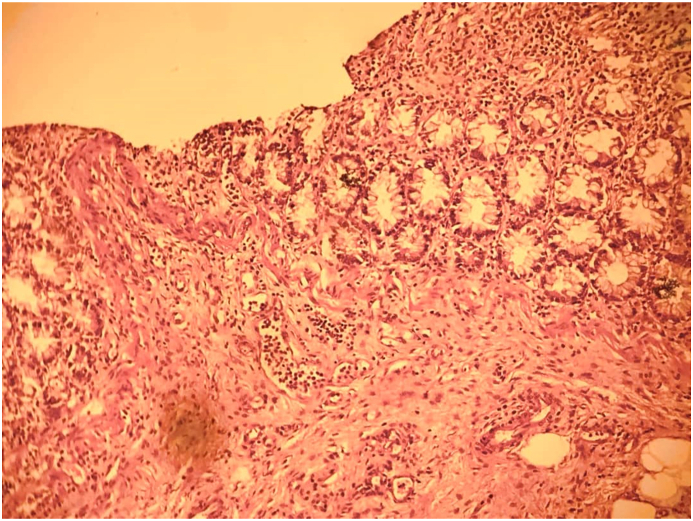
Photomicrograph of colon diverticula showing acute on chronic inflammatory changes (H&E straining, X100).

**Fig. 3:**
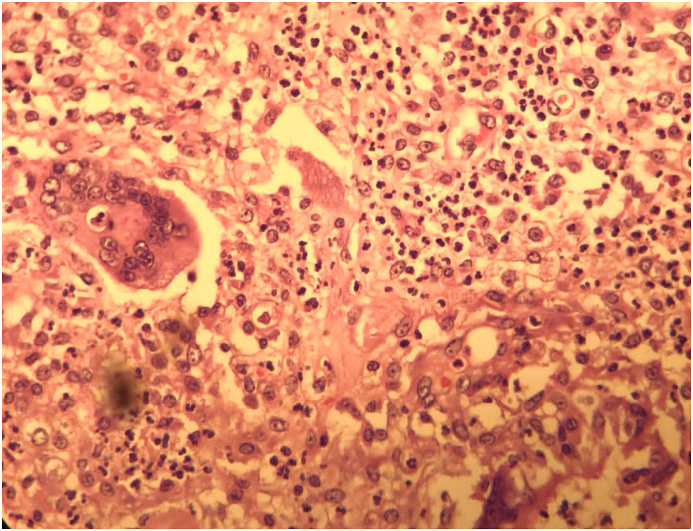
Photomicrograph of fallopian tube suppurative foreign body type granulomatous inflammation (H&E straining, X100).

Pelvic inflammatory disease (PID) refers to acute and subclinical infection of the upper genital tract, involving whole or parts of uterus, fallopian tubes, and ovaries, which is often accompanied by the involvement of neighboring pelvic organs. TOA (tubo-ovarian abscess) can be a complication of PID, which is an inflammatory mass involving the fallopian tube, ovary, and, occasionally, other adjacent pelvic organs (bowel, bladder). This may manifest as a tubo-ovarian complex (an agglutination of those structures) or a collection of pus (TOA). TOA typically occurs as a complication of PID but in some cases, is developed due to pathology of adjacent organs or hematogenous spread [1](Fig. 1, Fig. 2, Fig. 3). TOA requires aggressive medical and/or surgical therapy since rupture of an abscess may result in sepsis [2]. TOA is presented with fever and chills (not all cases) along with nausea, vaginal discharge and vaginal bleeding [3]. PID is diagnosed by the means of pelvic imaging, advised in patients who are acutely ill, no-to-poor response to antibiotic therapy, adnexal mass noted on examination and/or significant abdominopelvic tenderness precluding a complete pelvic examination [4].

Diverticular disease of the colon is an important cause of hospital admissions that is suspected in patients with lower abdominal pain (typically in the left lower quadrant), abdominal tenderness on physical examination, and leukocytosis. The diagnosis is usually confirmed by an abdominopelvic computed tomography (CT) scan. Acute complicated diverticulitis (such as frank perforation, obstruction, fistula, abscess) requires treatment of both, colonic inflammation (diverticulitis) and the specific complication which typically requires hospitalization and/or surgery [5,6]. Abscesses occur in 16–40% of patients with complicated acute diverticulitis. The overall success rate of non-operative management for diverticular abscess is about 80% [7]. The remaining 20% needs surgical intervention [8,9].

In this case report, we discuss a case of TOA patient who underwent surgery due to lack of response to pharmacological treatment and intra operative finding showed complicated diverticulitis.

## 2 Case report

45 years old woman G1P1 (gravida 1, parity 1) was admitted in emergency room with the history of chronic abdominal pain for two months, which was exacerbated two weeks ago. Her pain was colicky in the LLQ (left lower quadrant). The patient had no gastrointestinal and urinary symptoms. Her menstruation was normal without any history of vaginal discharge and dysmenorrhea. However, she complained of dyspareunia, fever and chills. In previous ultrasound finding, a heterogeneous mass of adnexal origin measuring 60*46 mm in the form hemorrhagic cyst was reported along with adjacent inflammatory changes and mild ascites. Second ultrasound after 3 weeks, performed due to exacerbation of pain, reported similar heterogeneous cystic mass measuring 71*52 mm with loculated fluid adjacent to the iliac fossa, in favor of peduncle degenerative myoma.

**Fig. 4:**
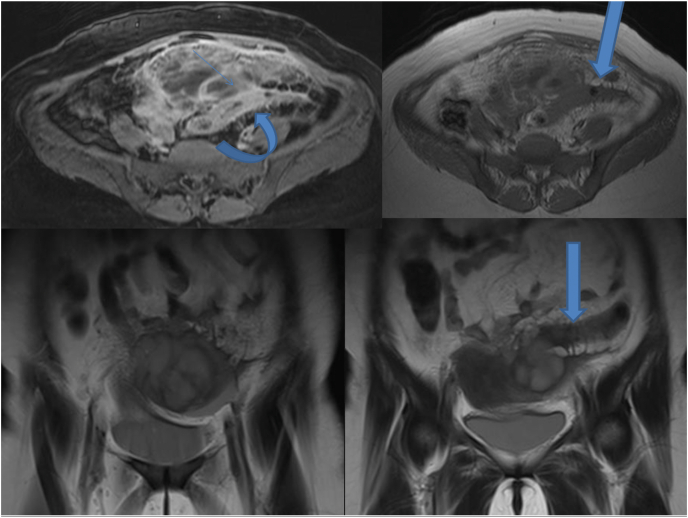
In post contrast T1 fat suppression image thick wall sigmoid colon(curved arrow) is detected just adjacent mass adnexal complex mass, focal area of obliterated fat plane between the adnexal structure and anterior sigmoid colon wall is seen (thin arrow), likely site of connection between these two structures. T1 axial, T2 sagital images, complex left adnexal mass with surrounding fat infiltration and thick left colon wall(arrow).

**Fig. 5:**
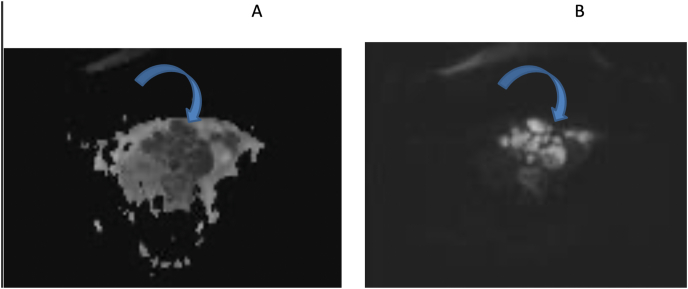
DW and ADC pelvic images, restricted adnexal complex due to abscess formation) curved arrow).

At the time of admission, the patient's vital signs were: blood pressure: 110/60, heart rate: 114 and body temperature 37.1 °C. On physical examination, the abdomen was soft and had tenderness on the left side of the abdomen along with palpable mass. Gynecological examination showed a firm tender mass in the midline measuring 16 weeks pregnant uterus. The patient was hospitalized with a possible diagnosis of degenerative myoma. Her laboratory findings were WBC: 17500, Hb: 9.8, MCV: 69, pregnancy test: neg, CRP: 187, ESR: 125, CA125: 26.9, CEA: 2.23. Ultrasound showed mixed echo mass measuring 79*44*57 mm with debris and echogenic line of about 150 cc in favor tubo-ovarian abscess or gastrointestinal mass. MRI also showed the evidence of tubo-ovarian abscess indicated by the loss of flat planes between the mass and adjacent pelvic organs and multiseptated lesions with pronounced air-fluid. Thus, ampicillin, clindamycin and gentamicin were started, which was switched with imipenem due to no change in abscess size, and a catheter was placed under ultrasound guidance to drain the abscess, for 5 days (Fig. 4, Fig. 5). During the hospitalization, due to leukocytosis and elevated CRP and ESR, evaluation for COVID-19 was performed and cardiac counseling was requested owing to unexplained tachycardia without any fever. Her hematocrit and hemoglobin were normal and was hemodynamically stable. During follow-up, the size of the abscess was decreasing, and leukocytosis was improving therefore the antibiotic was continued for two weeks. A follow-up ultrasound was performed that showed a large lesion. Due to recurrence, patient underwent laparotomy where multiple adhesions were seen. After emptying and removing abscess and releasing the adhesions, another lesion was seen on the left side of the abdomen that was surrounded by perforated sigmoid, uterus and ovaries, which was full of fecaloid fluid. The sigmoid was completely fixed with ovary wall and cyst was seen. After releasing the adhesions, a hysterectomy and a left oophorectomy was performed along with sigmoidectomy and ileostomy. Pathology revealed multiple sigmoid diverticula and fistulized ovaries along with acute inflammation and the presence of multinuclear giant cells.

There were no major postoperative complications and patient was discharged in healthy condition.

This case report has been reported in line with the SCARE 2020 criteria [10]. Written informed consent was obtained from the patient for publication of this case report and accompanying images.

## 3 Discussion

In this case study, we report a patient with acute diverticulitis where symptoms and adnexal mass was seen with the involvement of gynecological organs. Sigmoidal involvement in gynecologic diseases is also possible. Although rare, the sigmoid could be involved in abdominal pregnancies [11].

We searched PubMed database, Embase and Cochrane library using “diverticulitis”, “gastrointestinal complication”, “tubo-ovarian abscess” and “pelvic inflammatory disease” as keyword and excluded the cases where diverticulitis was presented as gynecologic complication. We found few cases that reported this complication. The mean age of patient was 43 (range:15–80 years). 9 patients were presented with abdominal pain and the duration between abdominal pain and diagnosis ranged from 3 days to 1 year. 5 cases were presented with fever, 2 cases had vaginal discharge, a patient had dyspareunia and dysmenorrhea. 5 patients had gastrointestinal symptoms such as diarrhea, constipation and vomiting whereas urinary tract symptoms were not reported. Medical history of these patients showed gastrointestinal disease such as irritable bowel syndrome, Crohn's disease, diverticulitis and Henoch–schonlein purpura. Common lab findings were leukocytosis in 7 patients, elevated CRP in 2 cases and elevated ESR a case. TOA was differential diagnosis in all the cases and mass-like lesion was seen in radiography. Involvement of sigmoid colon was reported in 5 cases and the most common involvement was edematous wall due to inflammation and mesentery fat thickening whereas evidence of diverticula was seen in two cases only. Drainage and broad-spectrum antibiotic for two weeks were the course of treatment in 3 studies, whereas, exploratory laparotomy was performed in the rest. The removal of abscesses wall included salpingo-oophorectomy, hysterectomy and colectomy. Overall, 7 cases of TOA complicated with diverticulitis (1 appendix diverticulitis and 2 sigmoid) were seen [[12], [13], [14], [15], [16], [17], [18], [19], [20], [21]].

Usually, pelvic abscess in women is attributed to genital organ and other causes are ignored. However, common cause of TOA is genital organ complication, especially in sexually active female [22]. Gastrointestinal disease is usually the most common causes of pelvic abscess that can involve genital organ. Diverticular disease of the colon is an important cause of hospital admissions, and due greater prevalence, the complications are more common. In our case and patients of the cases reviewed, diverticulitis was the most common causes of gastrointestinal manifestations. This complication revealed symptom similar to those of genital tract. Therefore, it can be like TOA, without fever and leukocytosis. Furthermore, due to the lack of inflammatory sign and proximity to genital organ, it can misdiagnosed as genital diseases/anomalies for example ovarian cysts, tumoral lesion, pedunculated or degenerated myoma [23]. Treatment of TOA and complicated diverticulitis is similar. Antibiotic and noninvasive procedure followed by surgical intervention is performed among patients who do not respond to pharmacological therapy [1,6,19]. Intraoperative findings and severity of disease determine surgical plan.

## Ethical approval

All procedures performed in this study involving human participants were in accordance with the ethical standards of the institutional and/or national research committee and with the 1964 Helsinki Declaration and its later amendments or comparable ethical standards.

## Sources of funding

No funding was secured for this study.

## Author contribution

**Dr. Seyyedeh Neda Kazemi:** conceptualized and designed the study, drafted the initial manuscript, and reviewed and revised the manuscript. Dr. Majid Samsami and Dr. Hamidreza Didar: Designed the data collection instruments, collected data, carried out the initial analyses, and reviewed and revised the manuscript. Dr. Masoomeh Raoufi and Haniye Najafiarab: Coordinated and supervised data collection, and critically reviewed the manuscript for important intellectual content.

## Consent

Not applicable.

## Registration of research studies

1. Name of the registry: N/a.

2. Unique Identifying number or registration ID: N/A.

3. Hyperlink to the registration (must be publicly accessible): N/A.

## Guarantor

Seyyedeh Neda Kazemi.

## Provenance and peer review

Not commissioned, externally peer-reviewed.

## Declaration of competing interest

The authors deny any conflict of interest in any terms or by any means during the study.
